# Case Report: Early Recognition, Treatment, and Occupational Safety Protection are Crucial for Methanol Toxicity

**DOI:** 10.3389/fmed.2022.918812

**Published:** 2022-06-14

**Authors:** Xiaomei Wu, Meifeng Gu, Wei Wang, Hainan Zhang, Zhenchu Tang

**Affiliations:** ^1^Department of Neurology, The Second Xiangya Hospital, Central South University, Changsha, China; ^2^Hunan Key Laboratory of Tumor Models and Individualized Medicine, The Second Xiangya Hospital, Central South University, Changsha, China

**Keywords:** occupational exposure, methanol poisoning, prevention, fireworks factory, sequela

## Abstract

**Background:**

Despite significant progress in treating methanol poisoning, the lack of training, hazard communication, and occupational safety protection education contributes to the risk of occupational exposure and methanol toxicity. In addition, early diagnosis and timely medical care are essential to reduce the risk of morbidity and mortality, yet it remains a challenging procedure.

**Case Report:**

A 35-year-old man working in a fireworks factory came to our emergency department with acute mental change and progressive disturbance of consciousness. The patient's vital signs were stable, and he presented with enlargement of both pupils with a weak reaction to light. Head computed tomography showed low signal intensities in the bilateral basal ganglia. He was admitted to the neurologic intensive care unit, where additional laboratory workup showed high anion-gap metabolic acidosis. Methanol poisoning was thus considered. Before being treated with sodium bicarbonate infusion, hemodialysis, folate, and high-dose vitamin B, the blood and urine samples were collected for toxicity tests, which turned out to be methanol poisoning. After 8 hours of hemodialysis, the patient's consciousness recovered, but he complained of a complete loss of vision in both eyes. Brain and optic nerve magnetic resonance images showed bilateral symmetric putamen lesions and optic neuropathy. Ophthalmic tests indicated visual pathway impairment and optic disc swelling but no fluorescein leakage. The right eye's vision was partially restored on the third day, but he could only count fingers at 20 cm. Unfortunately, his eyesight ceased to improve during the 6 months of follow-up.

**Conclusions:**

Early diagnosis and prompt treatment will improve the prognosis of methanol poisoning in terms of vision and patient survival. Awareness and supervision of commercial alcohol use are indispensable for similar industrial processes.

## Introduction

Methanol poisoning usually occurs when alcohol-dependent individuals consume low-cost household products, such as solvents, antifreeze, or fuel. As a result of occupational exposure, methanol poisoning is rarely reported (1). Methanol is well absorbed through the gastrointestinal tract, skin, or inhalation. While methanol is non-toxic, its metabolites (formaldehyde and formic acid) are highly toxic and can cause blindness, coma, metabolic disorders, and even life-threatening (2). Despite advances in the treatment of methanol poisoning, it remains one of the leading causes of death caused by poisoning due to the challenging diagnosis and late hospital admissions (2). Clinicians should remind that the triad of high anion gap metabolic acidosis, acute mental change, and vision disturbances suggests methanol poisoning (3). Surviving patients may suffer permanent visual impairment (4). Here, we report a case of early recognition and treatment of methanol poisoning in which the patient's symptoms improved significantly, and his vision was partially restored after treatment.

## Case Presentation

**Figure 1:**
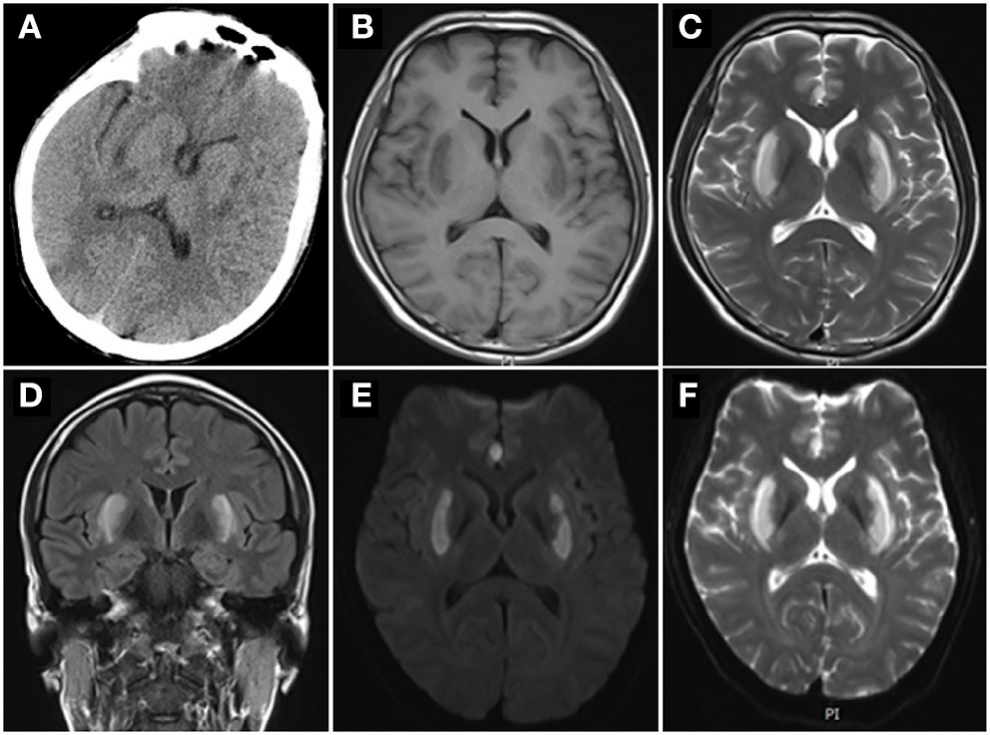
Brain images. **(A)** CT scan showed bilateral symmetric basal ganglia low density. Brain MRIs showed low signal intensities in the bilateral symmetric putamen in T1 **(B)**, and high signal intensities in T2 **(C)**, Axial FLAIR **(D)**, diffusion weighted image **(E)**, apparent diffusion coefficient **(F)** over the same regions.

A 35-year-old man was admitted to our emergency department with unexplained acute mental change, shortness of breath, and progressive disturbance of consciousness for 20 h. It is worth noting that he has prolonged exposure to alcohol and acetone for working as a firework maker for 8 years. His vital signs were stable and normal at first. Then he was admitted to the neurologic intensive care unit due to his rapidly worsening mental status to sopor (Glasgow Coma Scale E3V2M4 = 9). Physical examination revealed slurred speech, enlargement of both pupils (6 mm) with poor pupillary reflex responses, and more marked on the left side. Initial blood gas measurements revealed metabolic acidosis: pH 7.20 (normal range, 7.35 to 7.45), PCO_2_ of 10 mmHg (normal range, 35 to 45 mmHg), bicarbonate level of 3.8 mmol/L (normal range, 21 to 25 mmol/L), anion gap of 33.4 mmol/L (normal range, 8 to 18 mmol/L), and lactic acid level of 3.16 mmol/L (normal range, 0.60 to 2.20 mmol/L). CT scan showed low signal intensities in bilateral basal ganglia (Figure 1A) and pulmonary infection. Due to these findings, methanol poisoning was considered, and blood and urine samples were promptly collected for toxin analysis. Then the patient received treatment with sodium bicarbonate infusion, hemodialysis, folate, and high-dose vitamin B. In addition, he had a blood ammonia level of 98.3 umol/L (normal range, 16 to 60 umol/L) and an β-hydroxybutyric acid level of 1.31 mmol/L (normal range, 0.03 to 0.3 mmol/l). He had a total leukocyte count of 26.92 × 10^9^ cells/L (normal range, 3.5 × 10^9^ to 9.5 × 10^9^ cells/L), blood interleukin 6 level of 296 pg/ml (normal range, 0–7 pg/ml), blood procalcitonin (PCT) level of 6.44 ng/ml (normal range, 0–0.05 ng/ml), which indicated infection. Thus, antibiotics were initiated.

**Figure 2:**
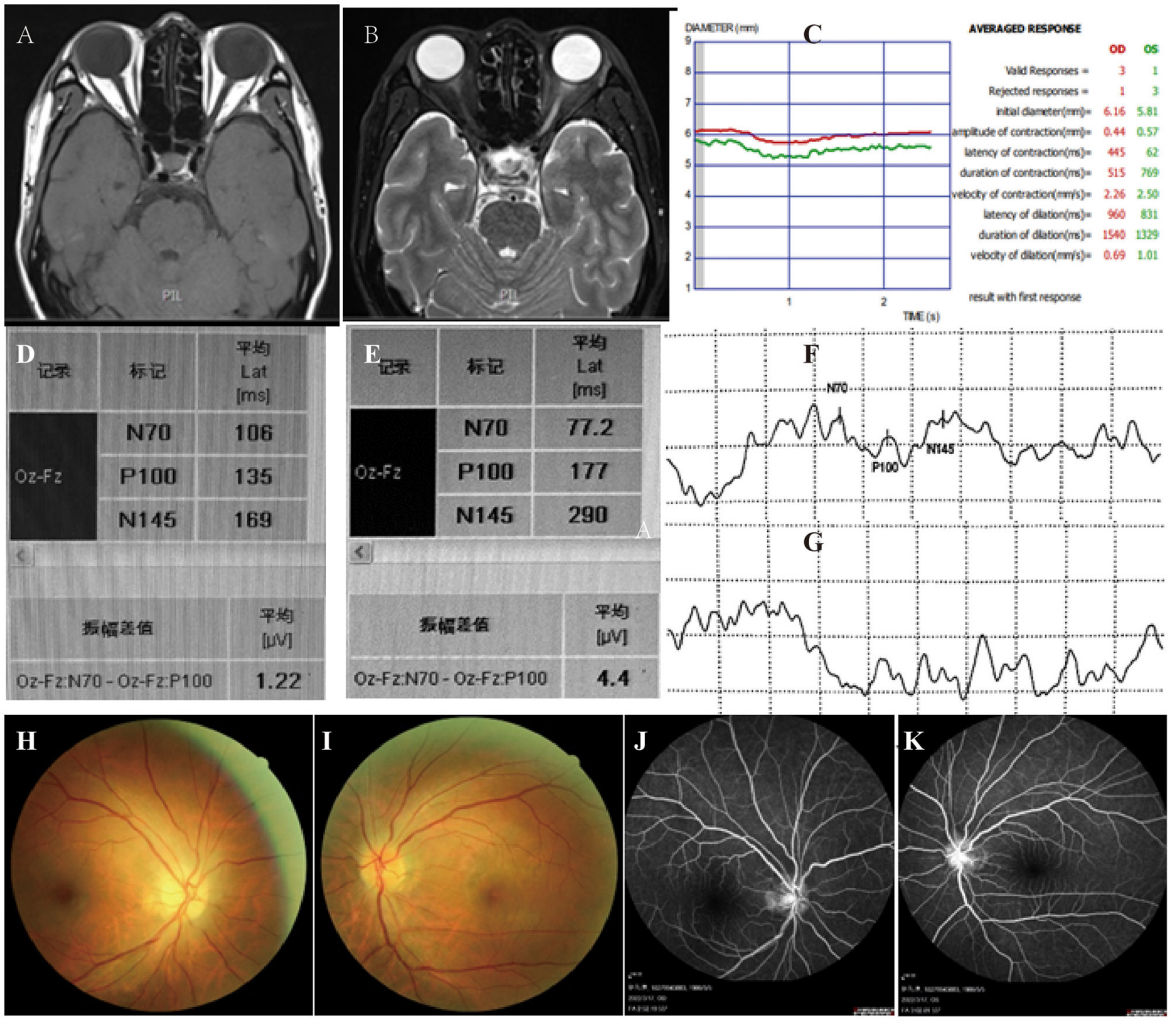
Optic nerve MRIs: T1 **(A)** and T2 **(B)** showed increased signal of bilateral optic nerve, especially the left one with thickening. **(C)** Pupillary light reflex showed both eyes are insensitive to light. Visual evoked potentials demonstrated prolonged latent time of P100 and decreased amplitude of wave in the right eye **(D,F)**, and no clear waveforms of P100 were elicited in the left **(E,G)**. Fundus examination revealed bilateral optic disc swelling **(H,I)**, especially the right one **(H)**. Fundus fluorescein angiography demonstrated no fluorescein leakage **(J)** and **(K)** for left and right respectively).

After 8 h of hemodialysis, the patient's consciousness recovered, and blood gas measurements were normal. Bicarbonate infusion and hemodialysis were ceased. However, his vision examination showed no light perception bilaterally. The serum and urine toxicology from initial samples confirmed methanol poisoning (details in Table 1). Due to the leukocytosis and high PCT indicating infection, we performed the para-eyeball corticosteroid injection instead of high dose steroids intravenous. Furthermore, we took a detailed history from the patient. He had long-term exposure to alcohol and acetone because of his job in a fireworks manufacturing factory without an organic-vapor cartridge or other air-purifying respirators, but nothing went wrong previously. He did not wear a mask or gloves as usual, and he took a hot bath right after work. A few hours later, he felt moody and uncomfortable with shortness of breath and unable to retrieve memories. Therefore, we conclude that the patient was occupationally exposed to a mixture of methanol absorbed by inhalation and transdermal route. Brain magnetic resonance images (MRIs) demonstrated abnormal signal intensity in the bilateral symmetric putamen (Figures 1B–F). A Series of examinations related to eyes were conducted. Optic nerve MRIs illustrated optic neuropathy (Figures 2A,B). Pupillary light reflex showed that both were insensitive to light (Figure 2C). Visual evoked potentials revealed visual pathway impairment, and optic nerve demyelination with axonal deterioration, which was more severe on the left side (Figures 2D–G). Fundus examination revealed features of toxic neuropathy with bilateral optic disc hyperemia and swelling (Figures 2H,I), and fundus fluorescein angiography showed no fluorescein leakage (Figures 2J,K). We performed the intermittent hemodialysis once more to remove residual methanol and its metabolites based on the toxicology result. Meanwhile, intravenous methylprednisolone 500 mg once a day for 5 days was commenced with oral folate 10 mg three times and thiamine 100 mg daily. On day 3, the patient seemed well, and his visibility improved to counting fingers at 20 cm in his right eye, but no light perception in the left eye. After 10 days on the ward during steroids tapering, the patient was discharged from the hospital and continued oral thiamine 100 mg and methylprednisolone 60 mg daily—the latter for 2 weeks. During the subsequent visit of 6 months, his eyesight ceased to improve.

**Table 1:** The methanol and formate concentration change in blood and urine after treatment.

**Date**	**Blood concentration (mg/L)**		**Urine concentration (mg/L)**	
	**Methanol**	**Formate**	**Methanol**	**Formate**
Before IHD	223	254	495	6428
After first IHD	54.5	53.5	43.8	3094
After second IHD	<1	3.1	<1	14.9
After third IHD	<1	<1	<1	10.1

*IHD: intermittent hemodialysis*.

## Discussion

It is well known that methanol poisoning most commonly occurs after ingestion of low-cost household products (2). Occupational exposure, although rare, may also occur *via* inhalation and transdermal absorption (3), resulting in severe neurological deficits. Despite strides made in the treatment of methanol poisoning, it remains highly fatal due to challenging diagnosis and delayed treatment (4). Even patients who survive may suffer permanent visual impairment (5). In our case, the patient had only a 24-h interval between the onset and hemodialysis and was left with visual impairment despite prompt treatment. Therefore, early diagnosis and appropriate treatment are critical for improving patient prognosis.

Confirmation of diagnosis relies on positive serum methanol or formate assay. While the existing techniques, gas or liquid chromatography, is laborious, expensive, and often unavailable (6). Familiarity with methanol poisoning patients' clinical presentations and major laboratory features is crucial for diagnosis. The clinical presentation of our patient started with shortness of breath, followed by progressive mental changes with progressive consciousness impairment. Moreover, laboratory tests suggesting severe metabolic acidosis were a vital hint. In addition, a history of exposure to toxic alcohol was particularly relevant. Many documents have mentioned that clinicians should consider methanol poisoning when encountering the triad of visual disturbances, high anion gap metabolic acidosis, and mental disorders. Methanol is non-toxic, but its metabolites (formic acid, methanol) are incredibly toxic. The main toxic effects do not become apparent until methanol is metabolized to formic acid and accumulates to toxic levels. Therefore, clinical symptoms typically evolve within 6 to 24 h but can be postponed to 72 to 96 h if ethanol is co-ingested (7). Initially, the patient became drowsy, unsteady, and out of control, and is often unnoticed. After a variable time, headache, vomiting, abdominal pain, and vertigo accompanied by shortness of breath may attack the victims. Visual impairment is one of the most common neurological symptoms ranging from small scotomas to permanent blindness (8). In the acute phase of poisoning, congestion, and inflammation of the optic disc might be observed. However, high anion gap metabolic acidosis is always a crucial clue (9). The acidosis that occurs early in methanol poisoning is due to the accumulation of formic acid (10). Subsequently, formic acid inhibits mitochondrial cytochrome-c oxidase and inhibits cellular respiration and the Na^+^-K^+^ ATPase pump, leading to increased anaerobic respiration and lactate production (11). Both formic acid and lactic acid contribute to increased anion gap and acidosis in methanol toxicity.

**Figure 3:**
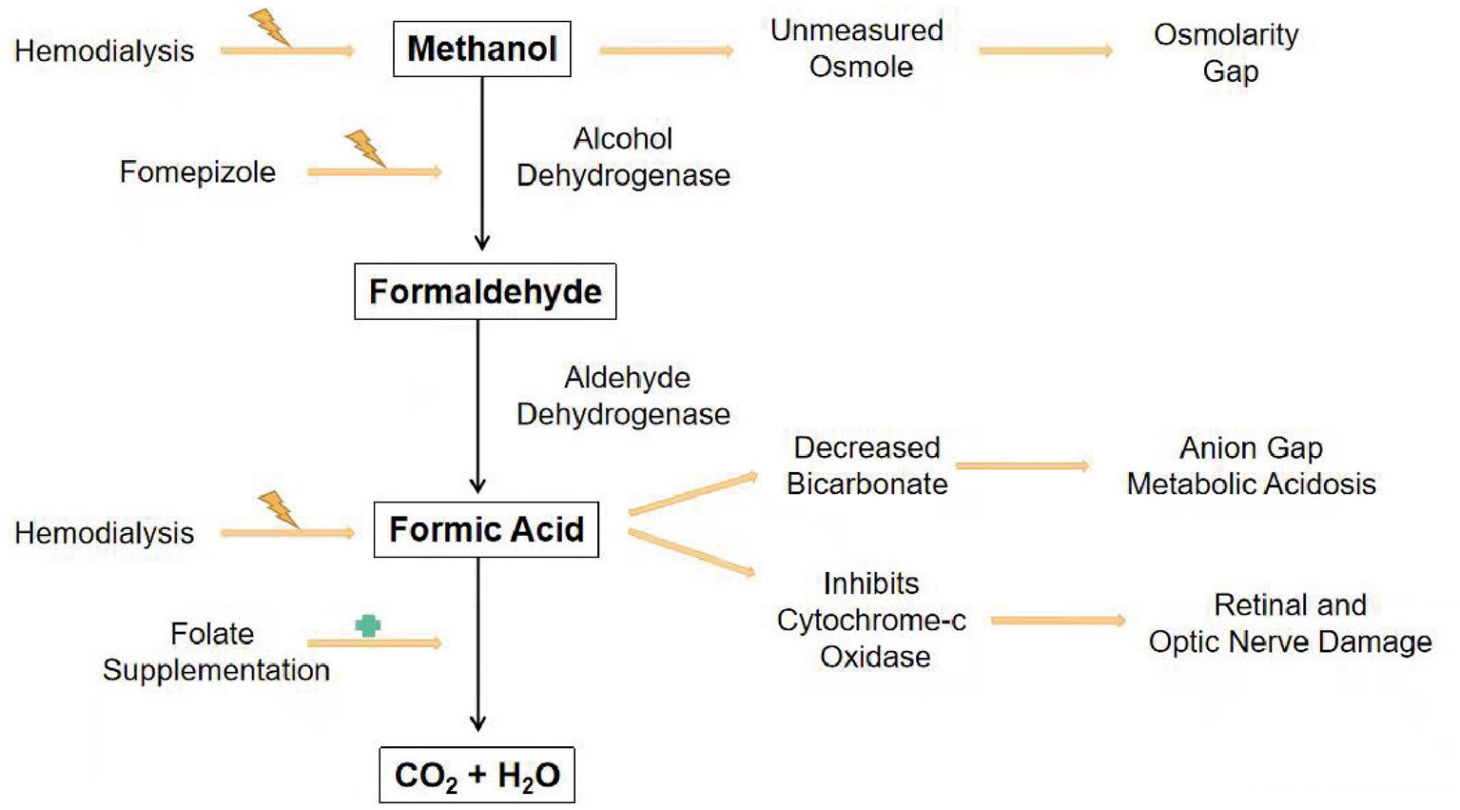
Simplified diagram of the methanol pathway with the resulting signs and interventions.

As for methanol treatment, gastrointestinal absorption of methanol is so rapid that stomach decontamination is generally not practical. The primary methods are prevention of metabolism and elimination of methanol and its metabolites from the body (3, 4, 12–14). Figure 3 shows the simplified diagram of the methanol pathway with the resulting signs and interventions. Sodium bicarbonate infusion corrects metabolic acidosis and increases the ionization of formate, thereby promoting its urinary excretion and reducing its penetration into the optic nerve (15). Due to its strong affinity for alcohol dehydrogenase, ethanol, no matter oral or intravenous, is frequently used for treatment (16), but not recommended for central nervous system inhibited patients such as our case. Fomepizole is a more potent alcohol dehydrogenase inhibitor with 8,000 times affinity. Furthermore, it is effective at low concentrations with minimal side effects. The point, however, is often unavailable outside the United States. Hemodialysis removes small and water-soluble molecules from the blood. Thus, methanol, formaldehyde, and formic acid are easily removed by dialysis. Both guidelines recommend dialysis for patients with severe metabolic acidosis, serum methanol concentrations above 51 mg/dl (16 mmol/L), deteriorating despite supportive treatment, vision problems, or acute kidney injury (4, 17). Treatment is expected to continue until the serum methanol concentration is <19–29 mg/dl (6–9 mmol/L) (17). Furthermore, intermittent hemodialysis removes methanol faster than continuous renal replacement therapy (17, 18). In patients with methanol poisoning, folate promotes the conversion of formic acid into carbon dioxide and water.

Methanol poisoning usually causes severe and permanent visual impairment. The histopathology of patients with methanol intoxication showed demyelination in optic nerves (19). A high dose of steroids might benefit the methanol-induced optic neuropathy (20, 21). However, early treatment with steroids remains debatable due to the possibility of causing immunosuppression and increasing the likelihood of secondary sepsis. Considering that our patient might be affected with sepsis due to the high level of PCT and pulmonary CT scan, we chose para-eyeball corticosteroid injection at first. And then, intravenous methylprednisolone was performed when the infection was under control. After a therapy series, the patient progressed from blind to finger-counting at 20 cm. However, methanol toxicity is a rare disease, and there is no high-level evidence to support the use of steroids. Moreover, more studies are required to identify the usage of steroids.

Last but not least, our case did not get appropriate protective equipment nor adequate methanol protection education, leading to an acute onset after a hot bath. And public health, as “the science and art of preventing disease, prolonging life and promoting health through the organized efforts and informed choices of society, organizations, public and private, communities and individuals” (22), is of great importance in occupational disease prevention. Much attention should be focused on methanol poisoning prevention due to its severe consequences. We suggest strengthening occupational health knowledge training of workers to highlight self-protection awareness and intensify individual protection. Workers should be trained in the safe operation process and equipped with personal safety equipment such as chemical safety glasses, antistatic workwear, rubber gloves, and anti-toxic masks before being exposed to methanol. The employers should pay attention to ventilation and detoxification equipment, and the methanol concentration in the air or adhesive should be monitored regularly. Most importantly, relevant departments and authorities should conduct on-site supervision and organize a comprehensive investigation of fireworks production enterprises.

## Conclusion

On the one hand, early diagnosis and prompt treatment are essential for patients with methanol poisoning. On the other hand, awareness and supervision of commercial alcohol use are indispensable for similar industrial processes. Thus, reinforced supervision of commercial alcohol use and training, risk communication, and personal protective equipment may improve the situation for the risk of occupational exposure. Much remains to be done, from occupational exposure prevention to improving the outcome of methanol poisoning.

## Data Availability Statement

The raw data supporting the conclusions of this article will be made available by the authors, without undue reservation.

## Ethics Statement

The studies involving human participants were reviewed and approved by the Ethics Committee of the Second Xiangya Hospital, Central South University. The patients/participants provided their written informed consent to participate in this study. Written informed consent was obtained from the individual(s) for the publication of any potentially identifiable images or data included in this article.

## Author Contributions

XW edited the manuscript for important intellectual content. MG collected the materials, conducted the follow-up, and drafted the manuscript. WW took part in the follow-up part. ZT assessed the patient, performed the review of the literature, and drafted the manuscript. HZ provided this case and edited the manuscript for important intellectual content. All authors contributed to the article and approved the submitted version.

## Conflict of Interest

The authors declare that the research was conducted in the absence of any commercial or financial relationships that could be construed as a potential conflict of interest.

## Publisher's Note

All claims expressed in this article are solely those of the authors and do not necessarily represent those of their affiliated organizations, or those of the publisher, the editors and the reviewers. Any product that may be evaluated in this article, or claim that may be made by its manufacturer, is not guaranteed or endorsed by the publisher.
