# Corrigendum: Case report: early recognition, treatment, and occupational safety protection are crucial for methanol toxicity

**DOI:** 10.3389/fmed.2023.1304395

**Published:** 2023-11-28

**Authors:** Xiaomei Wu, Meifeng Gu, Wei Wang, Hainan Zhang, Zhenchu Tang

**Affiliations:** ^1^Department of Neurology, The Second Xiangya Hospital, Central South University, Changsha, China; ^2^Hunan Key Laboratory of Tumor Models and Individualized Medicine, The Second Xiangya Hospital, Central South University, Changsha, China

**Keywords:** occupational exposure, methanol poisoning, prevention, fireworks factory, sequela

In the published article, there was an error in Table 1 as published. The unit of methanol and formate concentration is wrong. It should be mg/L, not mg/dL in the Table 1. The corrected Table 1 and its caption “The methanol and formate concentration change in blood and urine” appear below.

**Table 1:** The methanol and formate concentration change in blood and urine after treatment.

**Date**	**Blood concentration (mg/L)**		**Urine concentration (mg/L)**	
	**Methanol**	**Formate**	**Methanol**	**Formate**
Before IHD	223	254	495	6428
After first IHD	54.5	53.5	43.8	3094
After second IHD	<1	3.1	<1	14.9
After third IHD	<1	<1	<1	10.1

IHD: intermittent hemodialysis.

The authors apologize for this error and state that this does not change the scientific conclusions of the article in any way. The original article has been updated.

